# A novel antiviral strategy by disrupting the equilibrium of virus-host calcium homeostasis

**DOI:** 10.1016/j.coviro.2025.101497

**Published:** 2025-11-10

**Authors:** Erwan Brémaud, Belinda L Spillings, Johnson Mak

**Affiliations:** Institute for Biomedicine & Glycomics, Griffith University, Gold Coast, QLD, Australia

## Abstract

Calcium (Ca^2+^) homeostasis is essential for cellular signaling and protein trafficking. Viruses are comparatively simple organisms that leverage the availability of host cellular mechanisms for viral replication, including the manipulation of cellular Ca^2+^ dynamics-related processes.

We review the dependence of HIV on cellular Ca^2+^ to promote viral replication, including stabilization of viral protein complexes in clinically latent HIV infected cells. We also discuss some of the general reliance of cellular Ca^2+^ homeostasis on viral propagation across several viruses. We discuss viral manipulations of cellular Ca^2+^ regulation for viral replication, specifically for viral assembly, complex stability, and disassembly. We close with an exploration of therapeutic opportunities via Ca^2+^ homeostasis disruption to mediate dysregulation of viral complex formation for viral peptide antigen presentation. Thereby, we aim to unlock a novel strategy to achieve an antiviral effect by modulating host-cell regulatory processes.

## Introduction

**Figure 1:**
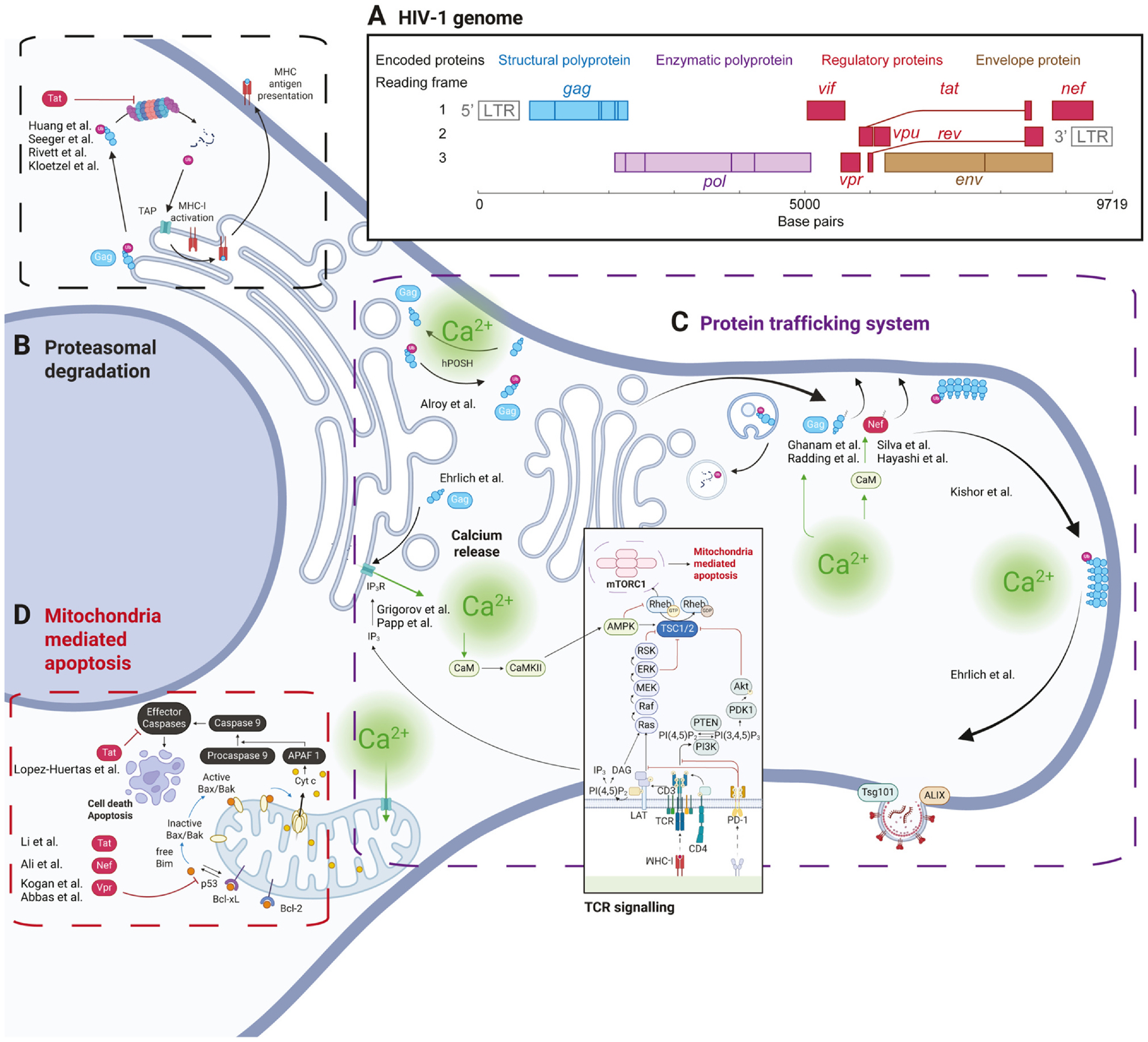
HIV-1 hijacks cellular Ca^2+^ homeostasis for protein complex formation and undercover replication. **(a)** Schematic describing HIV-1 genes and encoded structural, enzymatic, envelope and regulatory proteins. Multiple regulatory and structural HIV proteins influence Ca^2+^ release mechanisms to: **(b)** prevent antigen presentation, **(c)** favor viral protein trafficking from perinuclear synthesis locations to the uropod for assembly and budding, and/or **(d)** block apoptosis pathways for latency maintenance.

**Figure 2:**
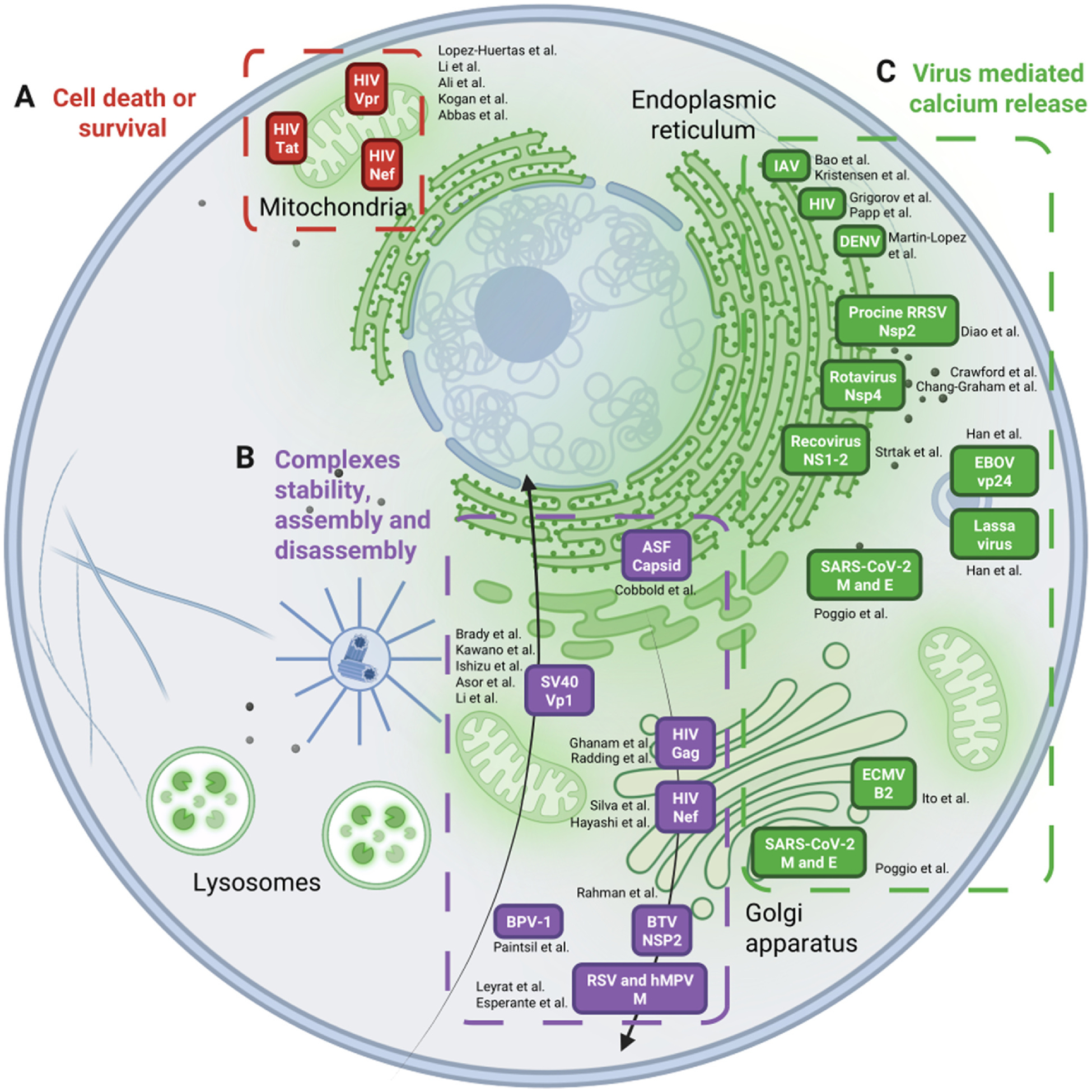
Hijacking of calcium metabolism is conserved across multiple viruses. **(a)** Viral proteins achieve control on calcium dependant mitochondrial apoptosis pathways. **(b)** Infections’ influence on cellular calcium homeostasis dysregulation, through indirect observations, or via identification of viral proteins mediating Ca^2+^ storage depletion. **(c)** Molecular characterization of Ca^2+^ involvement in protein complexes. Local fluctuations in calcium levels control complex stability, assembly or disassembly.

Clinical latency is the last major obstacle toward an HIV cure [1]. Direct cell–cell transmission has been highlighted as an important contributing factor for HIV immune evasion. In recent work [2], HIV-1 Gag assembly protein was shown to establish direct interactions with cellular Ca^2+^, contributing to directional membrane trafficking and viral assembly (Figure 1). Interestingly, impeding Ca^2+^ mediated viral protein interactions triggered increased viral protein ubiquitination, a marker of degradation, by destabilizing viral protein complex [2]. In this review, we explore a novel antiviral strategy through Ca^2+^ homeostasis disruption. By reviewing the breadth of Ca^2+^ related manipulations or reliance asserted by HIV (Figure 1), we highlight the observed importance of apoptotic drugs in HIV latency [3–7] is a likely consequence of the relationship between HIV and Ca^2+^ biology [2]. As Ca^2+^ homeostasis-related processes are essential for cellular metabolism [8], it is not surprising that several viruses hijack Ca^2+^-regulation-related machinery to replicate. This review compiles published literature describing the use or manipulation of cellular Ca^2+^ by viruses: (i) to enhance host cell survival to support virus production; promote viral replication; and (ii) to support viral protein trafficking for viral particle assembly (Figure 2). In the context of viral pathogens that lack effective antiviral countermeasures or protective vaccines, the broad spectrum of viral dependence on cellular Ca^2+^ biology could be leveraged for a new generation of antivirals. By exploiting Ca^2+^-virus relationship during viral replication, Ca^2+^ disrupting agents can be used to tip the balance to induce surface presentation of viral peptides for immune recognition and destruction.

## Maintenance of HIV involves cellular calcium

### A new perspective against HIV latency

While antiretroviral therapies (ART) effectively suppress detectable viremia and transmissibility between human hosts [9], HIV cannot be eradicated in people living with HIV (PLWH) under ART. Clinical latency remains the major obstacle for achieving a cure against HIV [1] and necessitates a new approach to the development of antiviral solutions. The relationship between ART, HIV reservoirs, and host immunity could be viewed as an equilibrium within PLWH. Here we explore a novel strategy to tackle HIV latency and induce clinical benefits to PLWH by tipping the equilibrium between HIV replication and the cellular processes HIV relies on.

Cell−cell transmission of HIV is a key mechanism for HIV to spread within the host and the maintenance of HIV reservoir [10]. Despite the efficiency of ART, a rapid rebound in viremia [11] is observed in ART-treated patients upon treatment interruption. The viral rebound is likely fueled by the continued, low level, cell–cell transmission within the lymphoid tissue with suboptimal local ART concentrations during treatment [12], also contributing to HIV invisibility to the host immune system [10,13]. In the context of Ca^2+^ biology, building upon HIV synapse biology and ubiquitination mediated quality control of cellular protein complexes, it is appreciated that Ca^2+^ release events from storage organelles promote the progression of secreted proteins from perinuclear synthesis to the plasma membrane [14,15]. Early works have reported that HIV leverages an overlapping *trans*-Golgi- / ubiquitin-protein ligase system for plasma membrane targeting, allowing HIV assembly and release [16]. Recent works show that HIV leverages Ca^2+^ fluctuations to achieve uropod membrane targeting [2], and the disruption of HIV–Ca^2+^ interaction increases the level of Gag ubiquitination that is earmarked for degradation [2]. Manipulation of host proteasome by viruses to limit viral antigen presentation is commonly utilized across viruses to propagate [17–19], including HIV via its transactivating (Tat) protein [20] (Figure 1a, b). The intimate relationship between viral pathogens and ubiquitination in quality control of protein, therefore, represents an unexpected strategy for the development of antiviral countermeasures.

### Calcium relationship with HIV replication

Besides Ca^2+^ role in directional targeting of HIV complexes via virological synapse [2] for cell–cell transmission, HIV depends on Ca^2+^ release from storage organelles (such as endoplasmic reticulum [ER] or mitochondria) to promote HIV production [21]. Activation of inositol 1,4,5-triphosphate receptor (IP_3_R) on ER for Ca^2+^ efflux results in enhancement of HIV particle release, while suppression of IP_3_R prevents particle production [21] (Figure 1c). A relationship between HIV and Ca^2+^ is further supported by the use of ionomycin [22], thapsigargin, or cyclopiazonic-acid [23] to encourage virus egress. Biophysical analyses show that truncated HIV assembly protein Gag can interact with Ca^2+^ binding protein calmodulin (CaM) with nanomolar (~10^−9^ M) affinity [24], and it is postulated that the HIV–CaM interaction facilitates, in part, the plasma membrane anchoring of HIV protein for assembly (Figure 1c). HIV negative factor (Nef), another HIV protein, also interacts with CaM [25] and is presumably associated with plasma membrane targeting (Figure 1c). Unlike the Ca^2+^-Gag relationship [2,21], Nef reduces local plasma membrane Ca^2+^ influx [26] to suppress immunological synapse function between CD4^+^ T-lymphocytes and antigen-presenting cells, thereby impairing T-cell-receptor (TCR)-mediated stimulation [27]. Mechanistically, Nef exploits the interconnectivity of Ca^2+^-associated vesicle traffic [26] and TCR signaling to re-route lymphocyte-specific protein tyrosine kinase away from the plasma membrane to limit immunological synapse response, thereby enhancing HIV spread as a consequence [28]. These ‘opposite’ and ‘local’ requirements of Ca^2+^ for HIV-Gag and -Nef to support viral replication illustrate the delicate balance for HIV proteins to coordinate and support HIV propagation via Ca^2+^ reliance and manipulation. Consequently, this precise balancing act that HIV must exert to regulate the host cell Ca^2+^ homeostasis also stands as an ‘Achilles heel’ for HIV replication and pathogenesis.

### Mitochondria, apoptosis, and HIV

In addition to ER, mitochondria represent a major intracellular Ca^2+^ storage [29], and it is perhaps less appreciated that mitochondria-mediated apoptosis events are highly interconnected with Ca^2+^ dynamics [30,31]. The notion that HIV persists in infected cells by interfering with apoptosis has been described [32]. In this context, HIV possesses an arsenal of multipotent proteins, contributing to HIV replication and potentially toward HIV persistence. The HIV regulatory proteins Tat, Nef, and ‘viral protein R’ (Vpr) (Figure 1a) hijack the survival/apoptosis pathway through signaling that involves cytoplasmic Ca^2+^ signals. Both functional Nef [33] and low levels of Tat [34] correlate with early infection and inhibit the apoptosis initiator p53. Furthermore, low levels of Vpr in early infection serve to upregulate B-cell lymphoma-2 (Bcl-2) levels/down-regulate Bax expression [35,36], while small amounts of Tat are able to block caspase 10 to prevent apoptosis [37] (Figures 1d and 2a). If coordination of intracellular Ca^2+^ homeostasis post-HIV infection is important for HIV to survive and propagate, it is not surprising to see a cohort of HIV proteins work cooperatively to take control of (or interfere with) mitochondria-associated biology, thereby hijacking Ca^2+^ regulatory mechanisms to support HIV replication [38].

### HIV latency reversion and apoptosis targeting drugs

Given the linkage between Ca^2+^, apoptosis, and mitochondria [30], it is noted that multiple promising compounds being developed to facilitate HIV cure are directly or indirectly influencing Ca^2+^-mediated pathways of mitochondrial-associated apoptosis. First, pro-apoptotic compound AZD5582 shows transcription onset *in vivo* in latent HIV humanized mice and SIV rhesus macaques [39], with enhanced depletion of the viral reservoir when combined with Rhesus monoclonal antibodies [40]. Second, immune checkpoint modulators anti-PD1 (Nivolumab and Pembrolizumab) disrupt Ca^2+^ dynamics [41] and show promises for latency reversal *ex vivo* [5] and in PLWH [3–5], and treatments are associated with increased viral production, detected plasma vRNA, and an instance of CD8^+^ response [3–5]. Third, the antagonist of Bcl-2 apoptosis mediator (Venetoclax) also showed efficiency *ex vivo* in conjunction with CTL response [6,7], suggesting HIV antigen presentation could have derived from degradation of HIV proteins via disruption of the Ca^2+^-stabilized complexes [2]. Fourth, this strategy of Ca^2+^ homeostasis disruption in eliminating latently infected cells is likely to be applicable to other human retroviruses, such as Human T-lympho-tropic virus 1. Myeloid cell leukemia-1 (Mcl-1) is an antiapoptotic protein and a key regulator of Ca^2+^ homeostasis via mitochondria biology. Recent work has shown that co-administration of Mcl-1 and ART has the potential to eliminate HTLV-1 *in vivo* using an humanized mice HTLV-1 model [42], adding weight on Ca^2+^ homeostasis disruption as a strategy for HIV cure.

## Viral protein complex trafficking and degradation are mediated by calcium

This section highlights the breadth of viruses that utilize Ca^2+^-related cellular processes to further their replication, including Ca^2+^-dependent vesicle transport system [43], such as the endosomal sorting complex required for transport (ESCRT) [44], that is widely used by enveloped viruses to replicate. By interfering with the Ca^2+^ homeostasis to induce protein degradation and surface presentation of viral peptides for immune destruction, it will be possible to attain a similar functional outcome as predicted in synthetic proteolysis-targeting chimeras (PROTACs) [45,46] by leveraging the natural relationship between virus and host. Through dysregulating calcium balance, Ca^2+^ controls the formation of viral complexes, yielding ill-assembled particles, prone to proteolytic degradation.

### Mechanistic contributions of Ca^2+^ to viral complex stability

With a number of viruses, the functional contributions of Ca^2+^ to support viral replication have been better defined, including the mediation of protein complexes stability for viral-assembly and -disassembly.

For example, a classical Ca^2+^ binding motif, EF hand motif, has been identified within the NS2 protein in Bluetongue virus (BTV) from the *Sedoreoviridae*. Biophysical analyses show the role of Ca^2+^ in BTV NS2 protein is to facilitate NS2-oligomerization and -phosphorylation for the production and release of infectious viral particles [47] (Figure 2b). Furthermore, a role of Ca^2+^ mediated viral particle assembly is also seen with human metapneumovirus (hMPV) from the *Pneumoviridae*. Specifically, structural analyses show that Ca^2+^ stabilizes the intramolecular structure of hMPV matrix (M) protein to permit hMPV M protein dimerization, for infectious particle formation [48]. Similarly, Ca^2+^ appears to function in an analogous manner with the respiratory syncytial virus (RSV, from the *Pneumoviridae*) M protein to increase the thermal stability of RSV M protein folding and facilitate oligomerization of RSV M protein for viral assembly [49]. It is speculated that Ca^2+^ stabilizes RSV M protein via intramolecular interaction through Ca^2+^ coordination [49] (Figure 2b). In contrast, rather than intramolecular interactions observed in hMPV M [48] and RSV M [49], Ca^2+^ facilitates inter-molecular interaction of Simian Virus 40 (SV40, from the *Polyomaviridae*) capsid protein for particle formation [50,51]. Additional data further implicate that these Ca^2+^-based interactions are involved in viral entry, cytoplasmic trafficking [52], and virus disassembly [52–54] (Figure 2b).

These lines of evidence that Ca^2+^ contributes to the functional assembly of viral protein complexes illustrate that viruses have evolved to hijack and/or manipulate Ca^2+^ machinery in host cells.

### Viruses induce changes in local intracellular Ca^2+^ concentrations

In this section, we review how viruses utilize cellular Ca^2+^ to support their replication, plus explore some of the potential antiviral targets via Ca^2+^ homeostasis disruption. Direct evidence of a link between Ca^2+^ and arboviruses such as dengue virus (DENV, from the *Flaviviridae*) comes from insect vector (*Aedes aegypti*) [55], in which DENV interacts with insect synaptosomal-associated protein and Ca^2+^ transporter ATPase for viral replication [55]. With the requirement of mammalian-ESCRT and —exocytosis for assembly and release of many members of *Flaviviridae* [56], there is little doubt about the involvement of Ca^2+^ regulation in Flavivirus biology (Figure 2c). Implications of other viruses in cellular calcium homeostasis dysregulation include: (i) Ebola- and Marburg-viruses from the *Filoviridae*; plus (ii) Lassa- and Junín-viruses from the *Arenaviridae* [57]. Data suggest the viral matrix proteins from both the *Filoviridae* and *Arenaviridae* are involved in inducing increased cytoplasmic Ca^2+^ concentration, thereby promoting viral assembly and egress [57] (Figure 2c).

Several viruses have further evolved to encode for their own proteins (in the form of viroporins) to directly manipulate the local Ca^2+^ concentration and support replication [58–61]. These viruses include: (i) severe acute respiratory syndrome coronavirus (SARS-CoV-2) from the *Coronaviridae*; (ii) encephalomyocarditis virus (ECMV) from the *Prionaviridae*; (iii) rotavirus from the *Sedoreoviridae*; and (iv) recovirus from the *Caliciviridae*. For example, SARS-CoV-2 viroporin protein E and structural protein M have been shown to rearrange the contact sites of organelles to disrupt cytoplasmic Ca^2+^ levels [58] (Figure 2c). Specifically, levels of Ca^2+^ stored in ER were measured by mediating Ca^2+^ release with histidine and compared with control across cells expressing SARS-CoV-2-E, -M, or both proteins [58]. The study depicted reduced release in the presence of M and E, indicating lower Ca^2+^ content in the ER, which was further explained by Ca^2+^ efflux allowed by E protein using an *in vitro* system to measure Ca^2+^ current throughout a suspended lipid bilayer [58]. Although the effect of SARS-CoV-2-E and -M proteins on viral replication is still unclear, the high level of sequence conservation of protein E across beta-coronaviruses suggests that an evolutionary advantage in controlling Ca^2+^ during infection confers a significant advantage. Similarly, the viral proteins: ECMV 2B; rotavirus Nsp4; and recovirus NS1–2 have been recognized as viroporins [59–61] due to their abilities to manipulate local Ca^2+^ concentrations. Using fluorescent calcium dyes, cytoplasmic Ca^2+^ concentrations were proven to increase upon infection [59], and calcium chelation resulted in a dose-dependent decrease in inflammasome activation or viral titers [60,61]. Whilst the benefit of ECMV 2B activity is still unclear [59], both rotavirus Nsp4 and recovirus NS1–2 have been shown to localize in the ER membrane and disrupt Ca^2+^ homeostasis, thereby facilitating replication [60,61] (Figure 2c).

In addition to direct Ca^2+^ mediation through viroporins, viruses hijack cellular Ca^2+^ channels to promote replication. A good example is Nsp4; besides its viroporin function, the protein also activates cellular Ca^2+^ channels (Stromal interaction molecule1 in ER membranes and Orai1 in the plasma membrane) [62]. Interestingly, Nsp4 was also highlighted as a virulence factor, capable of releasing extracellular signals, leading to Ca^2+^ uptake by cells surrounding infection [63,64]. During influenza A virus infection (IAV, member of *Orthomyxoviridae*), immunohistochemistry analyses show colocalization of IAV with voltage-dependent calcium channels (VGCC) in infected pigs [65], implicating a role of Ca^2+^ channel for viral infection (Figure 2c), and evidence of sialylated VGCC-mediated hemagglutinin binding confirms Ca^2+^ is essential during viral entry in muscle cells [66]. Similarly, entry of SARS-CoV-2 [67] and Ebola virus [68] and disassembly of SV40 [54] are all promoted by two pore Ca^2+^ channels. Utilization of cellular calcium channels by IAV, rotavirus, SARS-CoV-2, SV40, and Ebola viruses validates the use of calcium channel blockers (CCB) to achieve antiviral effects. CCBs include (i) dihydropyridines, which showed success in controlling infections from cytomegalovirus (CMV) [69] from *Herpesviridae* or SARS-CoV-2 [67], and (ii) non-dihydropyridines (i.e. Verapamil, Diltiazem, or Tetrandrine) inhibiting CMV [69] and Epstein-Barr virus from *Herpesviridae* [70], IAV [66], SARS-CoV-2 [71], Ebola virus [68], and SV40 [54] replication.

Building upon evidence of Ca^2+^ directed stabilization of viral complexes, and viruses hijacking cellular Ca^2+^ machinery to promote replication, this review introduces Ca^2+^ as a general viral mediator that can be exploited for the development of antiviral or countermeasures. The variety of examples described here highlights the breadth of Ca^2+^ dependency for viral replication, hence a large scope for clinical applications, specifically with viral pathogens that lack effective treatment or preventive vaccines.

## Conclusion

Decades of HIV research have confirmed a strong viral-host interdependence relationship with cellular calcium. Ca^2+^ supports viral assembly and trafficking mechanisms underpinning cell–cell transmission, thereby contributing to the maintenance of the viral reservoir. Our recent discovery [2] that the impediment of HIV–Ca^2+^ interactions-induced HIV protein ubiquitination lays the groundwork for Ca^2+^ homeostasis disruption as an antiviral strategy discussed here. Promising pre-clinical evidence [3–7] with Ca^2+^ homeostasis disruptors on HIV latency studies supports the idea that HIV-host Ca^2+^ equilibrium dysregulation could be a viable target. In this review, examples of Ca^2+^ dependency are highlighted across diverse virus families, representing an important yet underexplored translational application. Sharing the same goal as in PROTAC strategy to promote degradation of viral proteins, disruption of Ca^2+^ homeostasis to induce degradation of viral proteins and surface presentation of viral peptides is a practical solution to boost the antiviral immune response. In the context of viral infections that lack potent antivirals or preventive, ‘tipping the balance’ on the viral reliance on cellular Ca^2+^-homeostasis stands as a feasible alternative to weaken the ability of viral pathogens to persist in humans by complementing the adaptative immunity of the host.

## Data Availability

No data were used for the research described in the article.
